# Cultural taboos as a factor in the participation rate of Native Americans in STEM

**DOI:** 10.1186/s40594-018-0114-7

**Published:** 2018-04-11

**Authors:** Deborah H. Williams, Gerhard P. Shipley

**Affiliations:** 1 Department of Environmental Science, Johnson County Community College, 12345 College Blvd., Overland Park, KS 66210 USA; 2 Indigenous and American Indian Studies Department, Haskell Indian Nations University, 155 Indian Ave., Lawrence, KS 66046 USA; 3 Lawrence, USA

**Keywords:** Taboo, Sacred, Native American, Indian, STEM

## Abstract

**Background:**

Native Americans are underrepresented in science, technology, engineering, and mathematics (STEM). We investigated whether having to violate cultural taboos might be a factor in the decisions of some Native Americans not to pursue STEM degrees. Many STEM faculty likely know very little about Native Americans’ historical experiences with an education system that has been used to forcibly acculturate them and so may not be aware of the threat many Native Americans perceive from curricula that claim cultural neutrality yet require Native Americans to violate strongly held cultural beliefs.

**Results:**

We reviewed the relevant literature, surveyed 96 students from 42 different tribes, and interviewed two STEM and two non-STEM faculty at Haskell Indian Nations University. We found that 50% of survey respondents generally observe tribal taboos, 38% would choose not to pursue a science major if they knew or suspected that doing so would require them to violate an important tribal taboo, and 67% would be more likely to take science classes if the science curriculum was more respectful of tribal taboos. The most problematic activities and animals encountered in laboratory classes include, in order of discomfort level, human dissection, human bodies, animal dissection, snakes, spiders, and lizards.

**Conclusions:**

Increasing Native American participation in STEM requires that their cultural concerns regarding STEM curricula be acknowledged and addressed. This is important for several reasons. First, Native Americans have the highest poverty rate of all racial/ethnic groups, while STEM graduates have higher employment rates and salaries than non-STEM graduates. Second, increasing diversity in STEM supports cognitive growth and critical thinking, benefits problem solving, and contributes to increasing productivity, creativity, and global competitiveness. Third, there is a long history of exploitation of Native Americans and their lands by scientists and engineers, so it is particularly important to increase Native American participation so that their interests are represented in these professions. Many Native Americans’ concerns can be proactively and reasonably accommodated to provide a more respectful and welcoming learning and working environment and increase their participation in STEM, to everyone’s benefit.

## Background

Native Americans are underrepresented in the STEM disciplines. In 2012, Native Americans (i.e., American Indians and Alaskan Natives) were 1.7% of the population (United States Census Bureau 2015) but accounted for only 0.6% of bachelor’s degrees, 0.4% of master’s degrees, and 0.2% of doctoral degrees in science and engineering (National Science Foundation 2015). Many reasons have been asserted for the low participation rate of Native Americans in STEM disciplines, including lack of exposure, lack of interest, lack of confidence, lack of a feeling of belonging, and lack of goal congruency. For example, one factor may be the mismatch between the Native culture’s emphasis on communal work goals and the non-communal culture of STEM (Smith et al. 2014). Native American students who succeed in college do so “in part because they stayed engaged in their programs of study, often by finding meaningful connections between tribal beliefs and practices and the values and goals of American colleges,” but it can be difficult to find such connections in the STEM disciplines (Smith et al. 2014, p. 414). Further, many Native Americans have STEM knowledge that is rooted in naturalist traditions and arrived at through direct experience, and so, they may feel alienated by approaches to STEM that are not grounded in direct experience and denigrated by a system that appears to assume they know nothing about STEM (Cajete 2000; Nelson-Barber and Estrin 1995). Efforts to overcome these obstacles and increase Native American participation in STEM include in-school mentoring and out-of-school science education experiences which present science within culturally relevant frameworks (Stevens et al. 2016).

Historically, the education system was used to forcibly acculturate Native Americans by removing them from their families and tribes, assigning them European-American names, prohibiting them from speaking their languages, and generally stripping away every aspect of Native American culture in an attempt to “kill the Indian in him, and save the man” (Grimley 2015, quoting Pratt, 1892). Particularly relevant to this paper, the eradication of “savage superstition” was an expressed component of “civilizing” Native Americans (Grimley 2015, citing Pratt, 1892). So although science is often characterized as being “universal” and transcending any particular culture, some see a hidden curriculum of acculturation and assimilation within an education system that disregards or dismisses the cultural identities of its students (Grimley 2015, pp. 3–5). Most STEM faculty likely know very little about Native Americans’ historical experiences with the education system and so may not be aware of the threat many Native Americans may understandably perceive from curricula that claim cultural neutrality while requiring them to violate their cultural beliefs and identities (Bulow 1991). If Native Americans feel they have to set aside their Native identity in order to participate in STEM, then many will simply not engage in STEM learning.

For many Native Americans, exposure, interest, and confidence may already be present, and it may not be only about a culturally relevant theoretical framework, but also about a culturally sensitive classroom and laboratory experience. “Although spirituality is often compartmentalized in mainstream discourse, it permeates Native culture” (Hodge and Limb 2010, p. 266). Institutions are legally required to reasonably accommodate all students’ sincerely held religious and moral beliefs, but accommodation is often in reaction to a request. Discussing taboos under such circumstances can be perceived as profoundly disrespectful (Hodge and Limb 2010), and many Native Americans may be inclined to altogether avoid situations requiring accommodation rather than enter those situations and request it. Thus, we investigated whether a factor contributing to the underrepresentation of Native Americans in STEM may be the reluctance of at least some Native Americans to violate cultural taboos, including profaning the sacred, with regard to certain animals, plants, and activities commonly encountered in undergraduate science laboratory classes.

## Methods

We defined “taboo” to mean a strong cultural warning or prohibition against an action, such that violating a taboo is an act of serious aberrance which can result in feelings of guilt or shame and/or direct or indirect social sanction. We defined “sacred” to mean a strong cultural veneration. The taboo and the sacred are often justified on moral or religious bases, but some may result from ecological and economic conditions. As Harris (1978) noted, “human society is neither random nor capricious. The regularities of thought and behavior called culture are the principal mechanisms by which we human beings adapt to the world around us” (p. 210), and categorizing things or activities as taboo or sacred is often a form of adaptation. We note that some acts of profaning the sacred are at least implicitly taboo, while other acts of profanity, though frowned upon, may not rise to that level.

We first reviewed the available literature in order to better understand the nature and scope of tribal taboos and their potential impact on Native American participation in STEM. There are currently 566 federally recognized Native American tribes and an additional number of state-recognized tribes and unrecognized tribes. We searched both popular and scholarly literature for Native American taboos and found that shockingly little scholarly work has been done to document them. We examined a large amount of literature purporting to broadly describe various Native American tribes and found very few accounts and no detailed treatment of taboos. We therefore limited our literature review of actual tribal taboos to the taboos of the two most populous tribes, the Navajo and the Cherokee, for which the most information is available, though much of that is found only on various websites maintained by the tribes, tribe-related groups, and individual tribal members. The results of the literature review, along with our own experiences taking and teaching STEM classes and taking and teaching classes with Native Americans, informed the design of surveys which we administered to a convenience sample of adult Native American students at Haskell Indian Nations University (HINU) in Lawrence, Kansas, in classes taught in the spring and fall semesters of 2017.

Survey respondents were asked to provide basic demographic information (e.g., tribal membership, age, sex). Respondents were then asked to answer “yes” or “no” to specific questions regarding their upbringing and education, their families’ and their observance of tribal taboos, and the impact of tribal taboos on their perception of and relationship with STEM. Lastly, respondents were presented with a list of animals, plants, and activities commonly used in science classes and asked to identify those that are taboo, and, for those so identified, select a discomfort level of “very,” “slightly,” or “not at all” if the respondents had to handle or be around the animals or plants or participate in the activities. In analyzing this data, we calculated the percentage of students who identified any particular item as involving a taboo, and we coded and summed their indicated discomfort levels to provide a better understanding of the overall importance and impact of each item. Recognizing that there may be more potentially problematic animals and plants than we identified, we asked respondents to identify other animals and plants the viewing, handling, or examination of which would violate a cultural taboo. The survey required approximately 30 min to complete.

From 125 potential respondents, 96 surveys (76.8%) were completed by members of at least 42 different tribes (with some respondents having connections to more than one tribe). Thirteen respondents identified wholly or partly as Navajo, making it the most highly represented tribe. Forty respondents were male, and 56 were female, and their ages ranged from under 20 years to over 40 years. While all HINU students are enrolled members of federally recognized tribes, it is possible that, by having already chosen to pursue postsecondary education, they may not be perfectly representative of their tribal populations, the majority of whom do not attend college.

We also conducted semi-structured interviews of two STEM and two non-STEM Native American faculty members at HINU. DHW conducted the interviews on the HINU campus at times and places convenient to the interviewees. A list of open-ended questions was prepared to spur discussion, but the interviewees were generally allowed to shape the conversations to better reflect their particular backgrounds and experiences with taboos and teaching Native American students.

The results of these three independent lines of evidence—literature review, surveys, and interviews—support and validate each other. We found no contradictions, and, in fact, we found very close concurrences between the animal, plant, and activity taboos discussed in the literature and the animal, plant, and activity responses on the surveys, and between the interviewed faculty’s answers and the surveyed students’ answers regarding the influence of taboos on Native Americans’ participation in STEM.

This study was approved by the Institutional Review Boards (IRBs) of the University of Kansas, HINU, and Johnson County Community College.

## Results and discussion

The results of the literature review are important to understanding and appreciating the results of the surveys and interviews. In particular, the taboos identified and emphasized in the literature were strongly reflected in the survey results. For example, taboos against dead bodies, especially dead human bodies, are strongly emphasized in the literature, and human and animal dissections were the most problematic STEM activities according to our survey respondents.

### Navajo animal and plant taboos

Bulow’s (1991) popular book is the primary source for Navajo taboos and is cited by Navajocentral.org, an unofficial website for the Central Navajo Nation, in its discussions of taboos. “In some ways almost all of the taboos are religious in nature since they are part of a right way of living” (Bulow 1991, p. 191). According to Hillerman, in his foreword to Bulow (1991), above all, “a Navajo must stay in harmony with the natural and supernatural worlds” (p. 9). The concept of harmony is reflected in the Navajo word *hozho* or *hozhoni* (“walking in beauty”). Attaining *hozho*, regaining it when it is lost, and ultimately perfecting it is the goal of Navajo ceremonialism and the center of Navajo philosophy; “the maintenance of *hozho* is the bedrock of the Navajo Way…” (1991, pp. 14–15). Violating taboos causes discordance, or sickness, and disrupts *hozho*, which must then be restored through appropriate ceremonies.

Snakes are the subjects of particularly strong taboos: “Do not kill snakes or lizards because it will make your heart swell, it will dry up, or you will get crooked teeth,” and “do not touch a snake because it has nothing and you will have nothing” (Bulow 1991, pp. 89, 93). Touching a snake will result in a *chein-dee* (“evil spirit”) entering the body and cause sores, illness, and aches and pains where the body touched the snake (Jannotta 1986). “Watching snakes eat is the same kind of thing. If you see a snake eat or even a picture of a snake eating, you will develop digestive problems” (Jannotta 1986). A zoo on the Navajo reservation removed a snake exhibit “because cultural beliefs about the reptiles as bad omens were deterring visitors from seeing other animals…Many teachers didn't want children seeing or even breathing the same air as the snakes” (Fonseca 2015).

According to Bulow (1991), “Owls, crows, mice, and coyotes are considered helpers of the witches and evil spirits” (p. 77). Rabbits are also associated with witches. Even though bear claws are commonly worn as talismans of power by many Native Americans, Navajos avoid all parts of the bear. Some insects are also the subjects of taboos. For example, killing grasshoppers will result in a nosebleed, killing moths will result in the offender jumping into a fire, and bees, grasshoppers, cicadas, and other insects are important in the creation story and other Navajo stories. “All water animals are the subject of taboo” (p. 77). For example, killing frogs, lizards, salamanders, and toads can result in so much rain as to cause a flood that will ruin crops; it can cause the offender to jump around, become crippled or paralyzed, become skinny, or bring disease; or it can negatively affect the offender’s unborn child. Horned toads are the grandfathers or guardians of arrowheads, and killing one will result in a stomachache, swelling, or a heart attack. “This is one of the taboos where Anglo biology teachers have a problem. They expect Navajo students to dissect frogs or other animals that live in the water. It is taboo for any Navajo to do anything with a dead animal, especially the ones associated with water” (p. 79).

Jannotta (1986) interviewed a teacher at a Navajo boarding school about her experiences teaching Navajo children without knowing much about their tribal customs:

I kept [a snake] in my classroom for the rest of the school year, and the kids asked me to bring it back the next year. I still have the same snake. Technically, I am continuing to break the social, or rather the religious, taboos; however, as a science teacher, I have found that many things I do break a taboo one way or another. Science and traditional beliefs do not usually coincide. Almost every place I go I am doing something that’s against the beliefs. I did a unit on instincts and had the kids try to make a bird’s nest after researching how birds build their nests. I was told afterwards that bird’s nests are taboo and that the same thing happens to you if you touch a bird’s nest that happens if you touch a snake. Bones are the same way. Owls are supposed to be representations of death; if there’s an owl around then it’s a messenger of death. The way I found out about that was doing a bulletin board with an owl on it…Everywhere I turn as a science teacher...I’m constantly running up against taboos. I have decided that I will take precautions in using certain things, for instance...the snake. I have it in a sealed terrarium with a big sign: Do Not Touch…I’ve had the younger adult community members come in and ask me things like, ‘What do you do in here? Do you do any dissections?’ I say no, we do none of that because it’s against the culture. They say, ‘Good, because I wanted to know if my child was going to be involved in something like that. That is very bad and we can’t let our children do that.’

With regard to plants, the Navajo hold sacred corn, squash, beans, and tobacco. “Navajos revere corn as a gift from the gods and invested it with great powers,” and in the creation story, First Man and First Woman were created from perfect ears of corn (Bulow 1991, p. 169). Further, “corn pollen is used in virtually every aspect of Navajo religious life and observance,” and one taboo states, “do not waste or play with corn pollen or you will have bad luck” (Bulow 1991, pp. 169, 172).

### Cherokee animal and plant taboos

According to Ketchum (2017), the Cherokee believe that “owls and other animals possess intelligent spirits and must exist alongside people in a harmonious and balanced fashion.” Owls are particularly spiritual in nature:

As owls were animal spirits of the upper world, some Cherokee shamans believed these birds–particularly Eastern screech owls, a common species in Cherokee territory–served as spiritual consultants on sickness and punishment…The owl's nocturnal nature and ghostly appearance sometimes earn it an association not only with sickness, but with death…The Cherokee sometimes endow the owl with a personality akin to that of a wise old man (Ketchum 2017).

Killing an eagle, wolf, or rattlesnake is forbidden (Ketchum 2017). With regard to plants, killing evergreens is generally avoided, and evergreen wood is never used for common tools or firewood (“Cherokee taboos” 2017). “Like the evergreens, ginseng, is a sacred plant and is respected. When seeking ginseng the first three or four plants are passed by, when the desired plant is found and uprooted with proper prayer some beads are placed in the hole. Any offering would really suffice but traditionally red beads are used for this” (“Cherokee taboos” 2017).

### Relevant activity taboos shared by both tribes

One of the strongest taboos common to the Navajo, Cherokee, and many other tribes involves interaction with dead human bodies. “Contact with dead human bodies means contact with evil…You go mad, become infertile, or die if you touch the dead” (Mathiasen 2006, p. 717), and “don’t touch a human bone [or else] the bad spirits will get you” (Bulow 1991, p. 201). Lori A. Alvord, the first female Navajo surgeon, said that her “ultimate challenge” in medical school involved human dissection: “Navajos do not touch the dead. Ever. It is one of the strongest rules in our culture” (Alvord and Pelt 2000, p. 40). She realized later that she might have requested accommodation to watch rather than actively participate, or to make use of computer simulations, but at the time, she felt she had no choice but to violate the taboo. She wrote, “One by one, I conquered the most difficult obstacles of medical school. But each step along the way felt like a step further from my own people and Native ways…The cost of my knowledge had been high” (Alvord and Pelt 2000, pp. 43, 58). More broadly, actual or perceived disrespect for any human biologic materials can be problematic. Frank Dukepoo, one of only a few Native geneticists, explained, “To us, any part of ourselves is sacred. Scientists say it’s just DNA. For an Indian, it is not just DNA, it’s part of a person, it is sacred, with deep religious significance. It is part of the essence of a person” (Petit 1998).

Among other common taboos, the anthropomorphization of certain animals can make some Native Americans uncomfortable. Bulow (1991) noted that “some of the [Navajo] taboos associated with bears are probably a result of their rather human appearance when they stand upright…The bear taboos all share a common motif, that if a human being mimics a wild animal he will become like that animal” (p. 95). Dorson (1955) told an interesting story involving “Smoky the Bear,” a character created by the US Forest Service to educate the public on preventing forest fires. Posters of Smoky the Bear were mutilated near the Sheshegwaning Indian Reserve in Canada. “In some cases the picture of the bear was torn out, leaving the message intact…Some Indians believe the bear-walker is a person who can appear in another form-animal, bird, or ball of fire and can put the curse of death on an enemy” (p. 57). Once the bear was replaced with a beaver, the mutilations stopped.

### General problem with conflicting origin narratives

STEM classes can also have trouble with conflicting origin narratives. For example, geneticists who assume that the Beringia hypothesis (which asserts that Native Americans are descended from Siberians who migrated across Beringia approximately 20,000 years ago) is widely accepted and largely settled science might be surprised to learn that many Native Americans strongly reject it in favor of their own origin narratives. Some suspect a political agenda behind the Beringia hypothesis to reduce Native Americans’ qualitatively different claim to the land (i.e., original inhabitants versus colonists) to a mere quantitative difference (i.e., earlier colonists versus later colonists). In fact, we have personally witnessed Native American students walking out of undergraduate and graduate classes in which the Beringia hypothesis was being discussed.

### Surveys of HINU students

As shown in Table 1, we surveyed 96 HINU students, including 40 males and 56 females, from 42 different tribes.

**Table 1:** Survey demographic information and responses to specific questions

Demographic information	Number
Participants	96
Male	40
Female	56
Different tribes	42
Responses to specific questions	% “yes”
Raised on reservation	54%
Attended reservation school	43%
Generally observe tribal taboos	50%
Parents or grandparents generally observe taboos	50%
Violating taboo in science class would cause difficulty with family member	30%
Would not major in science if doing so required violating taboo	38%
Would be more likely to take science classes if science classes were more respectful of taboos	67%

Fifty-four percent of our respondents were raised on reservations, and 43% attended reservation schools. Fifty percent generally observe tribal taboos, and 38% would choose not to pursue a science major if they knew or suspected that doing so would require them to violate a serious tribal taboo. Fifty percent indicated that their parents or grandparents are generally observant of tribal taboos, and 30% indicated that if they violated a tribal taboo in a science class, it would cause them difficulty with a family member. This indicates that even if some students are themselves not deterred by requirements to violate cultural taboos, they may be deterred by the potential harm to familial and tribal relationships. “Family, extended family, and social support are at the heart of traditional American Indian values. In addition, family support has been described as vital to American Indian educational persistence” (Flynn et al. 2012). Perhaps our most striking finding is that 67% of respondents agreed that if science classes were more respectful of tribal taboos, such as by avoiding them or providing alternatives, they would be more likely to take science classes. A substantially higher percentage of female respondents (74%) than male respondents (58%) answered this question affirmatively, which suggests there may be a gender aspect to this issue.

As mentioned, survey respondents were asked to, first, identify from a list those animals, plants, and activities that are taboo and, second, for those so identified, indicate a discomfort level if the respondent had to handle or be around the animals or plants or participate in the activities. The ten most problematic animals, plants, and activities are shown in Table 2, along with the percentage of respondents identifying them and their cumulative discomfort levels.

**Table 2:** Survey responses to specific animals, plants, and activities

Animal, plant, or activity	% identifying as taboo	Cumulative discomfort level
Human dissection	50	126
Human bodies	40	101
Animal dissection	41	94
Snakes	35	94
Spiders	23	54
Lizards	21	53
Horses	17	38
Frogs/toads	14	34
Corn pollen	14	31
Plant collection	15	28

With regard to animal dissection, one respondent commented that dead animals should be left alone to decompose in order to “breakdown and support future life” and further commented that if an animal is killed then every part of it should be utilized. Relatedly, several respondents commented that it is wrong to take from the earth without leaving something in its place or praying. This suggests that, beyond specific taboos, there may be a larger ethic at issue with regard at least to dissection and possibly to other teaching methodologies with similar approaches. Several respondents suggested that at least some of their concerns might be ameliorated by allowing students to perform appropriate ceremonies before and/or after handling such plants or animals or engaging in such activities.

As mentioned, recognizing that there may be more potentially problematic animals and plants than we identified, we asked respondents to identify other animals and plants the viewing, handling, or laboratory examination of which would violate a cultural taboo. Owls were identified by 50% of respondents as being associated with taboos. One respondent commented that their concern includes not only owl bodies but also owl feathers, pictures of owls, and generally, “anything involving an owl.” Among other animals, eagles, bears, and coyotes were frequently cited, and among plants, sage and tobacco were frequently cited.

### Interviews of HINU faculty

In discussing obstacles related to student participation in laboratory sciences, one HINU faculty member commented, “Based on my own observations and knowledge of many, certainly not all, but many traditions there would be issues that could very clearly preclude or pose obstacles to students wanting to participate in lab sciences.” One obstacle is objections to dissecting animals that might serve as clan totems or have significant importance in story-telling traditions as teachers. “This is something that would be taboo because of that individual’s relationship with that animal.” Even though Western scientists might note that the physical aspect of the animal is dead, and the physical aspect is all there is, for many Native people, the metaphysical aspect, the spiritual presence, of the animal is real and still very much present. “So I think there are Native people who would be very put off by that kind of activity, it could cause them tremendous stress, anxiety, because in their traditions this is not something that one would ordinarily engage in.” This faculty member shared the story of a Native American student who was attending a non-tribal college and was very enthusiastic about studying science, “but as they got into it and then began to ask if they could be excused from certain kinds of activities because of their own cultural values and traditions…at that particular school people were unwilling to be accommodating,” which caused the student to pursue another major. “They were led to believe that you can’t be a scientist unless you do these kinds of things, which I think is one of those challenges that scientists need to be very aware of…That’s one story, maybe among dozens, hundreds, where students because they were told they had to do this basically said ‘well, if I have to do this then I’m not going to be a scientist.’”

Another HINU faculty member reported an awareness of Native American students who would not take a science class because they knew or suspected that it would require them to violate a taboo. This faculty member further asserted having “always felt a blatant disrespect and disconnect of and for our non-human relatives [by STEM departments].” Recalling a field trip to a university museum where students encountered the mounted remains of Custer’s horse, Comanche: “The students were very upset by the display…it was terrible.” As a Native teacher and researcher, the faculty member told of encountering many of the same issues outside of HINU in which STEM departments expect a “mechanistic reductionist worldview.” “I find science to still be a bastion of white and male positionality and power, and I don’t find them willing to make space for different worldviews.” Another HINU faculty member in a non-STEM discipline noted that they also sometimes encounter the same or similar issues, including prohibitions on showing human remains (of, e.g., Kennewick man), and seasonal prohibitions on the retelling of creation stories. Both of these faculty members stated that they proactively avoid directly violating known taboos (e.g., by discussing but not showing specific human remains and generally discussing rather than specifically retelling sacred stories) and reactively accommodate additional concerns as they arise by flexibly adapting lessons to satisfy students’ concerns.

## Conclusions

Based on our personal experiences as graduate students and faculty, and on our review of the literature, we expected to discover that the possibility of being required to violate cultural taboos might be a factor in the decisions of some Native Americans not to participate in STEM. We also expected that HINU faculty members would confirm that greater respect for Native American culture would broadly improve the relationship between Native Americans and STEM and likely result in increased participation. However, we did not expect that, even among students who had already chosen to pursue postsecondary education, fully two thirds would be more likely to take science classes if the curriculum was more respectful of tribal taboos.

Improving Native American participation in STEM requires that students’ concerns be acknowledged and addressed, including allowing Native Americans to retain their cultural identities (e.g., Belgarde 1992; Flynn et al. 2012; Tierney 1992; Wright 1985). This is important for several reasons. First, Native Americans have the highest poverty rate of all racial/ethnic groups, while STEM graduates have higher employment rates and salaries than non-STEM graduates (Cataldi et al. 2014). Second, increasing diversity in STEM supports cognitive growth and critical thinking, benefits problem solving (Ferrini-Mundy 2013), and contributes to increasing productivity, creativity, and global competitiveness (Allen-Ramdial and Campbell 2014). As one HINU faculty member stated, “Scientific institutions should be seeking out Native students because they do bring something very different to the practice of science that students who do not have that different cultural lens may not be able to see. So Native students may indeed see and experience things that could be informative and productive to research that other students might not.” Third, there is a long history of exploitation of Native Americans and their lands by scientists and engineers, so it is particularly important to increase Native American participation in these professions so that their interests are represented in the cultures and goals of these professions (Cajete 2000). As we also discovered from interviewing faculty, once Native American STEM students become STEM teachers and researchers, it is important to continue to empower them to retain their cultural identities and to teach and perform research in a way that is relevant to them, their students, and their subjects.

Certainly, some taboos cannot be practically, ethically, or legally accommodated. For example, many Native American tribes have taboos regarding menstruating women, including separating them from the community, particularly men. Attempting to accommodate such a taboo would likely violate both privacy and anti-discrimination laws. Further, many taboos may not be reasonably accommodated without sacrificing necessary rigor and content. However, many of the most common taboos are easily addressed. Providing alternatives to handling or dissecting humans and animals, and removing owls, snakes, lizards, frogs/toads, and spiders from public display, and when possible, limiting their presence to specific rooms (dissection, avian, herpetology, and insect laboratories) would alleviate some of the strongest concerns of many of our respondents. In addition, allowing appropriate ceremonies to be performed prior to or following laboratory exercises might mitigate some problems with taboos. We recommend that individual STEM departments wishing to create more welcoming environments engage in dialogues with their local tribes. Native American educators and other leaders would likely be willing to participate in this effort, and proactively engaging them would further demonstrate openness and respect and could lead to increased collaborations and other mutual benefits. In this manner, many Native Americans’ concerns can be proactively acknowledged and accommodated to provide a more respectful and welcoming learning and working environment and increase their participation in STEM, to everyone’s benefit.

## Abbreviations

HINU: Haskell Indian Nations University
IRB: Institutional Review Board
STEM: Science, technology, engineering, and mathematics

## References

[CR1] Allen-Ramdial SA, Campbell AG (2014). Reimagining the pipeline: advancing STEM diversity, persistence, and success. Bioscience.

[CR2] Alvord LA, Pelt ECV (2000). The scalpel and the silver bear: the first Navajo woman surgeon combines Western medicine and traditional healing.

[CR3] Belgarde MJ (1992). The performance and persistence of American Indian undergraduate students at Stanford University (dissertation).

[CR4] Bulow E (1991). Navajo taboos.

[CR5] Cajete G (2000). Native science: natural laws of interdependence.

[CR6] Cataldi EF, Siegel P, Shepherd B, Cooney J (2014). Baccalaureate and beyond: a first look at the employment experiences and lives of college graduates, 4 years on (B&B:08/12) (NCES 2014-141).

[CR7] Cherokee taboos. Retrieved from http://www.aaanativearts.com/cherokee/cherokee-taboos.htm. Accessed 20 December 2017.

[CR8] Dorson R (1955). Indian bear taboo. Western Folklore.

[CR9] Ferrini-Mundy J (2013). Drive by diversity. Science.

[CR10] Flynn SV, Duncan K, Jorgensen MF (2012). An emergent phenomenon of American Indian postsecondary transition and retention. Journal of Counseling & Development.

[CR11] Fonseca, F. (2015). Navajo zoo cuts snake exhibit over tribal beliefs. Retrieved from http://www.azcentral.com/story/news/local/arizona/2015/06/12/navajo-snakes-beliefs-eliminates-zoo-exhibit/71149118/. Accessed 20 December 2017.

[CR12] Grimley V (2015). Native American students living in dual worlds: community based education and funds of knowledge (dissertation).

[CR13] Harris M (1978). India’s sacred cow. Human Nature.

[CR14] Hodge DR, Limb GE (2010). Conducting spiritual assessments with Native Americans: enhancing cultural competency in social work practice courses. Journal of Social Work Education.

[CR15] Jannotta, M. (1986). Navajo taboos and seventh grade science. Retrieved from http://castle.eiu.edu/scienced/329options/navajo.html. Accessed 20 December 2017.

[CR16] Ketchum, D. (2017). Cherokee beliefs about owls. Retrieved from http://sciencing.com/cherokee-beliefs-owls-5634.html. Accessed 20 December 2017.

[CR17] Mathiasen H (2006). The body in the dissection lab: thing or taboo. The American Journal of Medicine.

[CR18] National Science Foundation, National Center for Science and Engineering Statistics. (2015). Science and engineering degrees, by race/ethnicity of recipients: 2002–12. Detailed Statistical Tables NSF 15-321. Retrieved from http://www.nsf.gov/statistics/2015/nsf15321/. Access 20 December 2017.

[CR19] Nelson-Barber S, Estrin E (1995). Bringing Native American perspectives to mathematics and science. Teaching Theory into Practice.

[CR20] Petit, C. (1998, February 19). Trying to study tribes while respecting their cultures: Hopi Indian geneticist can see both sides. San Francisco Chronicle. Retrieved from http://www.sfgate.com/news/article/Trying-to-Study-Tribes-While-Respecting-Their-3012825.php. Accessed 20 December 2017.

[CR21] Pratt, RH. (1892). "Kill the Indian, and Save the Man": Capt. Richard. H. Pratt on the education of Native Americans. Retrieved from http://historymatters.gmu.edu/d/4929. Accessed 28 March 2018.

[CR22] Smith JL, Cech E, Metz A, Huntoon M (2014). Giving back or giving up: Native American student experiences in science and engineering. Cultural Diversity and Ethnic Minority Psychology.

[CR23] Stevens S, Andrade R, Page M (2016). Motivating young Native American students to pursue STEM learning through a culturally relevant science program. Journal of Science Education and Technology.

[CR24] Tierney WG (1992). Official encouragement, institutional discouragement: minorities in academe, the Native American experience.

[CR25] United States Census Bureau. (2015). 2011–2015 American community survey 5-year estimates. Retrieved from https://factfinder.census.gov/faces/tableservices/jsf/pages/productview.xhtml?src=CF. Accessed 20 December 2017.

[CR26] Wright B (1985). Programming success: special student services and the American Indian college student. Journal of American Indian Education.

